# Inflammatory Biomarkers and Neurotrophic Factors in Preterm Newborns as Predictors of Motor Development: A Systematic Review

**DOI:** 10.3390/pediatric18010007

**Published:** 2026-01-05

**Authors:** Letícia Silva Gabriel, Vicente Donisete Ferreira Júnior, Marina Ornelas Anastácia Pereira, Dayanne Gabriela de Melo Marques, Virgínia Mendes Russo Vallejos, Melina Barros-Pinheiro

**Affiliations:** 1 Campus Centro-Oeste Dona Lindu, Universidade Federal de São João del-Rei (UFSJ), Rua Sebastião Gonçalves Coelho, 400, Divinópolis 35501-296, MG, Brazil; 2 Faculdade de Fisioterapia, Departamento de Ciências da Reabilitação e Saúde, Universidade do Estado de Minas Gerais (UEMG), Campus Divinópolis, Divinópolis 35501-170, MG, Brazil

**Keywords:** preterm newborns, inflammation, motor development, neurotrophic factors, biomarkers

## Abstract

**Background/Objectives:** Preterm newborns (NBs) are at increased risk of motor developmental impairments. Evidence on inflammatory and neurotrophic biomarkers measured in the neonatal period as predictors of motor outcomes is scarce and heterogeneous. This systematic review synthesised data on inflammatory biomarkers and neurotrophic factors in Preterm NB as predictors of motor development (MD) up to 24 months of corrected age. **Methods:** MEDLINE, SciELO, Web of Science and Embase were searched for longitudinal observational studies of Preterm NB (World Health Organization definition) that measured one or more inflammatory biomarkers and/or neurotrophic factors in blood, urine or saliva and applied validated neurodevelopmental scales up to 24 months. Non-original reports, populations outside scope and studies with incomplete data were excluded. Methodological quality of primary studies was assessed with the Newcastle–Ottawa Scale (NOS). The protocol was registered in PROSPERO (CRD42022365839). **Results:** Of 1475 records, eight studies met the eligibility criteria. Higher neonatal concentrations of interleukin-6 (IL-6), interleukin-8 (IL-8), tumour necrosis factor-alpha (TNF-α) and C-reactive protein (CRP) were generally associated with poorer motor performance, although null findings occurred in some cohorts. One study assessing neurotrophic factors reported elevated urinary brain-derived neurotrophic factor (BDNF) and glial cell-derived neurotrophic factor (GDNF) among infants with below-expected MD. **Conclusions:** Inflammatory biomarkers show promise as early indicators of adverse MD in Preterm NB, but heterogeneity in populations, biospecimens, sampling windows, assays and outcome scales limits comparability and precludes definition of risk thresholds. Larger, standardised cohorts are needed to clarify the prognostic value of inflammatory and neurotrophic biomarkers and to inform early risk stratification.

## 1. Introduction

The classification of prematurity is based on the gestational age of the infant: moderate-to-late preterm (born between 32 and 37 weeks), very preterm (born between 28 and 32 weeks), and extremely preterm (born before 28 weeks) [1]. As indicated in a report published by the World Health Organization (WHO) in 2023, despite scientific advancements across various health sectors, the prevalence of premature births remains unacceptably high, with one in every ten babies born prematurely [2]. In Brazil, the prematurity rate remained approximately % between 2012 and 2019 [3], a relatively high ure in comparison to that observed in European countries [4]. Advances in perinatal and neonatal intensive care have substantially improved the survival of very preterm infants; however, many survivors remain at increased risk of long-term neurodevelopmental impairments, including motor deficits [5,6,7,8].

The aetiology of prematurity is multifactorial and involves several maternal risk factors, including physiological factors, medical history, age, lifestyle, genetics and socioeconomic and environmental conditions [5]. These characteristics have the potential to impact maternal and foetal health, ultimately leading to a range of clinical complications during pregnancy and resulting in preterm birth. This situation not only reduces survival rates but also increases the probability of sensory and attention deficits, motor development (MD) delays and learning difficulties, which negatively affect social integration and, consequently, quality of life [9].

Motor development refers to the progressive acquisition of gross and fine motor skills as the central nervous system matures [10]. Most newborns (NB) with typical motor development are able to achieve the expected gross and fine motor milestones, such as independent walking, pincer grasp and first words, between 12 and 18 months of age [11]. The use of motor scales for the assessment of NPMD represents a validated and reliable methodology for the measurement and monitoring of motor milestones during the initial years of life, as well as for the identification of potential developmental risks [7,8]. Widely used instruments include the Bayley Scales of Infant Development (second and third editions) and the Test of Infant Motor Performance (TIMP), which were also applied in several of the cohorts included in this review [12,13,14,15,16,17].

Motor scales are readily available and cost-effective instruments for assessing motor outcomes. However, the identification of clinical and social risk factors, early neuroimaging and neonatal screening tests—typically performed only in infants at clinical risk of motor delay—are also relevant. Moreover, the literature describes the evaluation of biomarkers as an adjunct resource that may assist in identifying an elevated risk of adverse neurological outcomes in preterm infants [18].

Recent studies have sought to demonstrate the relationship between inflammatory biomarkers and the adverse outcomes associated with prematurity [19,20,21]. These molecules are objective and quantifiable indicators that fluctuate in response to inflammatory occurrences, whether pathological or otherwise [20]. Higher systemic concentrations of pro-inflammatory cytokines during pregnancy and in the neonatal period have been linked to low birth weight, preterm birth and an increased risk of adverse neurological events in preterm infants [19,21]. Furthermore, inflammatory molecules and neurotrophic factors have been proposed as candidate biomarkers of neurodevelopment, including motor development, in preterm (NB) [20].

From a biological perspective, neonatal systemic inflammation has been implicated in white matter injury and subsequent neurodevelopmental impairments through mechanisms such as microglial activation, excitotoxicity and oxidative stress [20,21]. Conversely, neurotrophic factors such as brain-derived neurotrophic factor (BDNF) and glial cell line-derived neurotrophic factor (GDNF) play central roles in neuronal survival, synaptic plasticity and maturation of motor circuits, and altered concentrations in the neonatal period may reflect both injurious and compensatory processes with potential impact on later motor outcomes [8,16,20,22]. Previous systematic and integrative reviews have mainly focused on broader neurodevelopmental outcomes or combined preterm and term populations [7,20], and did not specifically synthesise evidence on inflammatory and neurotrophic biomarkers as predictors of motor development up to 24 months in preterm NB. This underscores the need for an updated and focused synthesis such as the present review.

In this context, we framed our research question using a PCC structure: Population—preterm neonates; Concept—inflammatory biomarkers, proteins of the acute phase, astroglial injury markers and neurotrophic factors measured in blood, urine or saliva; and Context—prediction of motor development outcomes up to 24 months of corrected age. Experimental and clinical evidence has linked systemic inflammation in the perinatal and neonatal periods to white matter injury and subsequent neurodevelopmental impairments in preterm infants [6,7,19,20,21,23]. Pro-inflammatory cytokines such as IL-6, IL-8 and tumour necrosis factor-alpha (TNF-α) may contribute to microglial activation, excitotoxicity and oxidative stress, thereby disrupting oligodendrocyte maturation, myelination and the organisation of motor pathways. In parallel, neurotrophic factors, particularly BDNF and GDNF, play essential roles in neuronal survival, synaptic plasticity and the maturation of motor and cognitive circuits, and altered concentrations of these factors in early life have been associated with neuromotor and cognitive delay in preterm and term populations [8,16,22]. Although systemic inflammatory mediators are sometimes described as “nonspecific”, they represent biologically plausible upstream signals of neuroinflammatory cascades in prematurity and are among the most frequently measured and feasible biomarkers in neonatal cohorts [6,24,25]. Thus, the objective of this study was to conduct a systematic review of neonatal inflammatory biomarkers and neurotrophic factors, including GFAP, in preterm NB as predictors of motor development.

## 2. Materials and Methods

This systematic review followed the methodological guidelines of the Cochrane Handbook for Systematic Reviews of Interventions [26] and was reported in accordance with the Preferred Reporting Items for Systematic Reviews and Meta-Analyses (PRISMA) statement [27]. The completed PRISMA 2020 checklist, indicating the page and section where each item is addressed, is provided as Supplementary Table S1 (Supplementary Materials). The protocol was prospectively registered in PROSPERO (CRD42022365839), and the review was conducted in accordance with the registered methods.

### 2.1. Electronic Search and Study Selection

The article searches were conducted by three independent researchers (VDFJ, MOAP, LSG) in the electronic databases MEDLINE (via PubMed), SciELO, Web of Science, and Embase. The search terms used were “premature,” “motor development,” “biomarkers,” and specific combinations with the included biomarkers (cytokines, interleukin-1 beta (IL-1β), interleukin-6 (IL-6), interleukin-8 (IL-8), interleukin-10 (IL-10), C-reactive protein (CRP), and tumour necrosis factor alpha (TNF-α), as well as neurotrophic factors: glial cell-derived neurotrophic factor (GDNF), and brain-derived neurotrophic factor (BDNF), and the prespecified biomarker glial fibrillary acidic protein (GFAP) (an astroglial injury marker). A comprehensive search strategy was applied to MEDLINE (via PubMed), Embase, Web of Science, SciELO and the Cochrane Library, without language or date restrictions. Complete, copy-pasteable search strings and all applied filters for each database are detailed in Supplementary Table S2. Biomarker-related keywords were restricted to inflammatory mediators/acute-phase proteins and neurotrophic/astroglial biomarkers prespecified in the protocol; therefore, biomarkers primarily reflecting oxidative/nitrosative stress or mitochondrial/energy metabolism dysfunction were not included as targeted search terms. The initial search was conducted in January 2022 and updated in July 2024. The updated search conducted in July 2024 used the same search strategy and screening procedures, with three independent reviewers and a fourth reviewer resolving any disagreements.

The eligibility criteria for the included studies were as follows: longitudinal observational studies published in scientific journals that measured at least one inflammatory biomarker and/or neurotrophic or the prespecified astroglial injury marker GFAP in blood, urine, and/or saliva in preterm NBs according to the WHO [1]. The studies were required to employ a validated scale for the assessment of neuropsychomotor development up to 24 months of life, with MD as the outcome. Additionally, the studies were required to report the sample size, means, medians, and measures of variability (standard deviation and standard error) for all evaluated groups. We restricted inclusion to observational cohort studies that followed preterm infants over time to assess motor development, as this design is the most appropriate for prognostic questions in which inflammatory exposures cannot be randomised for ethical reasons. The exclusion criteria included animal studies, editorials, letters, abstracts, studies with topics unrelated to the review objective, duplicates, populations differing from the proposed scope, and studies with incomplete data.

Three authors (L.S.G., M.O.A.P., and V.D.F.J.) independently reviewed the titles, abstracts, and full texts of eligible studies using Covidence (systematic review software; Veritas Health Innovation, Melbourne, Australia; web-based platform) Discrepancies were resolved by a fourth reviewer (D.G.M.M.). All eligible records, published in peer-reviewed scientific journals, regardless of language or publication date, were included in this review. Finally, a manual search of the references of eligible studies was conducted, and those that met the inclusion criteria were incorporated into the review. Reasons for exclusion at the full-text stage were prospectively recorded. A list of all 22 excluded full-text articles, together with the main reason for exclusion, is presented in Supplementary Table S3.

### 2.2. Data Extraction and Assessment of Methodological Quality

The data from the studies were extracted independently by three authors (L.S.G., M.O.A.P., and V.D.F.J.) with consultation from a fourth reviewer (D.G.M.M.) in the event of any discrepancies. The following information was extracted from each study: study design, author, year, sample size (*n*), birth weight in grams, gestational age in weeks, biological material collected and age at the time of collection, methodology used for biomarker analysis, and inflammatory biomarkers. The following were measured: inflammatory biomarkers (IL-1β, IL-6, IL-8,TNF-α, and CRP), neurotrophic factors (GDNF and BDNF), and the astroglial injury marker GFAP. Additionally, postnatal conditions that may trigger inflammatory processes were examined, as well as the neuropsychomotor development assessment scales (postnatal application time and classification scores according to each scale). Whenever available, we also recorded details of the biomarker assays, including the use of Meso Scale Discovery (MSD) multiplex electrochemiluminescence platforms, enzyme-linked immunosorbent assays (ELISA) and other immunoassay methods. Finally, a synthesis of the main results was provided.

To evaluate the quality of the studies, we employed the Newcastle–Ottawa Scale (NOS). This tool is used to evaluate the methodological quality of observational studies, including cohort and case–control studies. The NOS is structured around three primary domains of observational studies: participant selection, group comparability, and the outcome of interest [28,29]. The scale score for each study can range from 0 to 9, with those scoring ≥7 considered high quality, 5 to 7 moderate quality, and ≤4 low quality [28]. The evaluation was conducted by two independent reviewers (L.S.G. and D.G.M.M.), and in case of disagreement, a third reviewer (V.M.R.V.) made the final decision.

### 2.3. Quality Assessment of the Review

The methodological quality of this systematic review was appraised independently by two reviewers using the AMSTAR-2 (A MeaSurement Tool to Assess Systematic Reviews) instrument. Each of the 16 domains was rated as “Yes”, “Partial yes”, “No” or “Not applicable”, according to the original guidance. The AMSTAR-2 appraisal was conducted independently by two reviewers who are co-authors of this manuscript; disagreements were resolved by consensus. Detailed AMSTAR-2 ratings are provided in Supplementary Table S4.

### 2.4. Data Analysis

Data analysis followed the plan prespecified in the PROSPERO registration (CRD42022365839). We summarised study characteristics, biomarker panels, biospecimens, sampling windows, neonatal clinical context and motor outcomes using structured tables and descriptive synthesis. Given the anticipated clinical and methodological heterogeneity across cohorts, our primary approach was a narrative synthesis. We compared the direction and consistency of associations between neonatal biomarker levels and later motor outcomes, taking into account differences in gestational age, neonatal morbidities, biospecimens, assay methods and outcome scales. Quantitative meta-analysis was considered but ultimately not performed, because the small number of eligible studies and the variability in biomarker definitions, timing of measurements and outcome assessments did not allow for meaningful statistical pooling.

## 3. Results

### 3.1. Study Selection

**Figure 1:**
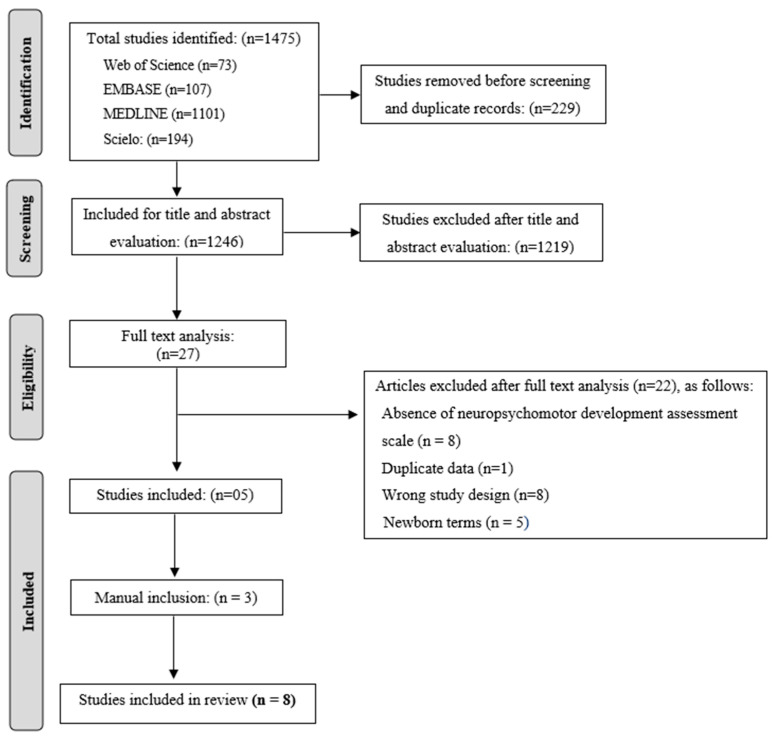
Flowchart of bibliographic search and evaluation of eligibility criteria.

A total of 1475 articles were identified through the database search. Following the removal of duplicates, 1246 records remained for screening. After evaluation of titles and abstracts, 27 studies were identified as potentially relevant and subjected to full-text analysis. Of these, 22 articles were excluded based on the following criteria: absence of validated motor development assessment scale and/or lack of motor outcome evaluation (*n* = 8), duplicate data (*n* = 1), study design not meeting the inclusion criteria (*n* = 8) and samples including only term NBs (*n* = 5). Three additional studies were identified by manual search, resulting in a total of eight articles included in this systematic review. The PRISMA flowchart for study selection is shown in Figure 1.

### 3.2. Methodological Quality of the Included Studies (NOS)

The quality of the included studies was assessed using the NOS (Table 1). Of the eight selected articles, three studies [12,13,30] scored ≥7 and were classified as “high quality.” The remaining five studies [14,15,16,17,31] were considered to be of “moderate quality,” with scores ranging from 4 to 6 points.

### 3.3. Quality Assessment of the Review (AMSTAR-2)

According to the AMSTAR-2 appraisal, the overall confidence in the results of this review was rated as high, with no critical weaknesses and a single non-critical weakness related to the lack of systematic extraction of funding sources from the primary studies (Supplementary Table S4).

### 3.4. Characteristics of the Included Cohorts

The eight studies included in this systematic review, published between 2011 and 2023, demonstrate notable variability with respect to the populations studied. Sample sizes ranged from small single-centre cohorts to large multicentre studies. O’Shea et al. [14] and Kuban et al. [12] included large cohorts of 939 and 439 preterm NBs, respectively, whereas Magalhães et al. [16] and Lee et al. [17] analysed smaller samples of 40 and 45 NBs. This variability in sample size may impact the precision and robustness of the findings, as smaller cohorts are more susceptible to random variation and bias (Table 2).

Gestational age also varied substantially across studies. Most cohorts analysed preterm infants with gestational ages ≤30 weeks, while O’Shea et al. [14] and Kuban et al. [12] focused exclusively on extremely preterm infants (≤28 weeks). Magalhães et al. [16] included NBs with a mean gestational age close to 30 weeks, and Lee et al. [17] investigated a broader range from 24 to 33 weeks. These differences are clinically relevant, as lower gestational age is a major risk factor for adverse neuropsychomotor development.

Most cohorts reported neonatal morbidities that could influence inflammatory status, such as late-onset sepsis, necrotising enterocolitis, chronic lung disease, ventricular enlargement, echolucent parenchymal lesions on cranial ultrasound, retinopathy of prematurity, acute disorders and death within the first three weeks of life [13,14,15,30,31]. In contrast, Magalhães et al. [16] did not report detailed neonatal morbidities, and the cohort reported by Nist M.D. et al. [31] explicitly excluded associated inflammatory conditions. Key neonatal morbidities and basic perinatal characteristics are summarised in Table 2.

None of the included studies consistently reported NICU length of stay in a way that allowed quantitative comparison between cohorts. For this reason, NICU length of stay is marked as “not reported” in Table 2.

### 3.5. Biomarker Sampling Windows, Biospecimens and Laboratory Techniques

With regard to the biological material analysed, the majority of studies collected blood samples to measure inflammatory and neurotrophic biomarkers. Magalhães et al. [16] additionally incorporated urine samples, exemplifying a more comprehensive approach to biomarker assessment. The use of different biospecimens may partly explain discrepancies in biomarker levels and their associations with outcomes.

The laboratory techniques used for biomarker measurement also varied among the studies. The MSD Multiplex platform was the most widely used, being applied in four studies [12,14,16,31] to allow simultaneous quantification of multiple biomarkers in a single sample. In contrast, other cohorts used enzyme-linked immunosorbent assays (ELISA) [17] or electrochemiluminescence-based methods [30], introducing methodological differences that may affect comparability of absolute concentrations. Absolute biomarker concentrations may differ across assay platforms (e.g., MSD multiplex vs. ELISA) due to variation in calibration standards and curve fitting, dynamic range and lower limits of detection, antibody specificity/epitope recognition, and matrix effects. In multiplex formats, additional variability may arise from cross-reactivity and reagent/analyte interactions, which can influence measured values. Therefore, cross-study comparisons of absolute concentrations should be interpreted with caution.

Regarding the biomarkers themselves, IL-6 was the most frequently assessed molecule, measured in seven of the eight included studies. IL-1β and TNF-α were also commonly evaluated, in five and six studies, respectively. However, neurotrophic factors such as BDNF and GDNF were analysed in only one cohort [16], indicating that investigation of these factors remains limited despite their potential relevance for motor development.

The timing of biological material collection also differed between studies. Silveira R.C. and Procianoy R.S. [15] did not provide details regarding the specific sampling times. O’Shea et al. [14] collected blood samples on days 1–3, 5–8 and 12–15 postnatal, whereas Rose et al. [13] collected samples within the first two weeks of life. Kuban K.C.K. et al. [12] obtained samples on days 1, 7 and 14 postnatal. Magalhães et al. [16] collected cord blood at birth and peripheral blood and urine at 48 h, 72 h and three weeks postpartum. Nist et al. [6] collected weekly blood samples until the infant reached 35 weeks of postmenstrual age. Lee et al. [17] obtained blood on the first day of clinical signs of systemic inflammation, and Kurul S. et al. [30] collected samples when sepsis was suspected, at least 72 h after birth. This diversity in sampling windows and biospecimens reflects substantial methodological heterogeneity across cohorts. Importantly, for cohorts in which biomarker collection timing was not reported in sufficient detail (e.g., Silveira) or was event-triggered (e.g., sampling at suspected sepsis), temporal interpretation of biomarker levels in relation to subsequent motor outcomes should be made with caution. Additionally, although several cohorts collected biomarkers at more than one neonatal time point, the included studies did not report sufficiently standardised and comparable longitudinal time-course data linking serial biomarker changes to repeated clinical neurological assessments and standardised motor scores over development. Therefore, our synthesis focuses on associations between biomarker levels measured within the reported neonatal sampling windows and motor outcomes assessed at predefined follow-up visits.

### 3.6. Motor Outcomes and Assessment Tools

Neuropsychomotor development was assessed using a variety of validated scales between 12 and 24 months of corrected age. Three studies employed the Bayley Scales of Infant Development, Second Edition (BSID-II) [12,14,15], while three others used the Third Edition (BSID-III) [13,17,30]. Two cohorts used alternative tools: the Test of Infant Motor Performance (TIMP) [16] and the Neurobehavioral Assessment of the Preterm Infant (NAPI) [31]. The timing of assessments and the scales applied in each cohort are summarised in Table 2.

### 3.7. Associations Between Inflammatory and Neurotrophic Biomarkers and Motor Development

Table 3 presents the main findings of the studies that evaluated the relationship between inflammatory biomarker levels and MD in preterm neurodevelopmental outcomes. Overall, there was considerable variability in the timing of biomarker measurement, in the periods during which NPMD was assessed and in the scales used for evaluation, as well as in the biomarkers assessed and in the co-occurrence of neonatal morbidities.

In the study by O’Shea et al. [14], preterm neonates who exhibited persistently elevated levels of inflammatory proteins in the blood during the first two postnatal weeks had a higher risk of severe developmental limitations at 24 months of life. Similarly, higher concentrations of IL-6, IL-8 and TNF-α, together with elevated CRP, were associated with less favourable motor performance in several cohorts [6,13,14,15,16,17,30]. In contrast, Kuban et al. [12] did not identify significant associations between these cytokines and the Psychomotor Development Index at two years of age, even in the absence of other reported inflammatory conditions, highlighting the complexity and non-linear behaviour of the inflammatory cascade.

The degree and type of inflammatory exposure also appeared to influence the direction and magnitude of associations. In Magalhães et al. [16], elevations in IL-1β and IL-8 during the initial days of life, measured in urine, were associated with typical motor development on the TIMP scale, in contrast to other cohorts in which higher cytokine levels predicted worse outcomes. Kurul et al. [30] did not find an association between IL-6 and MD delay in preterm infants with late-onset sepsis, possibly due to differences in timing, sample size, gestational age distribution and severity of sepsis.

Only one study evaluated neurotrophic factors. Magalhães et al. [16] observed that higher BDNF and GDNF concentrations were associated with worse motor prognosis among infants with below-expected motor performance, whereas external studies in larger populations have suggested that lower BDNF levels may serve as early markers of neurodevelopmental impairment [8,22]. Taken together, these findings suggest that both inflammatory cytokines and neurotrophic factors are promising, but context-dependent, candidates for risk stratification in preterm infants, as detailed in Table 3.

## 4. Discussion

This systematic review addressed whether inflammatory biomarkers, acute-phase proteins, astroglial injury markers and neurotrophic factors measured in the neonatal period among preterm infants are associated with motor or neuropsychomotor development up to 24 months of corrected age. Overall, the available evidence suggests that higher concentrations of pro-inflammatory cytokines (particularly IL-6, IL-8 and TNF-α) and C-reactive protein in the first weeks of life are associated with less favourable motor outcomes in most, but not all, cohorts. Evidence regarding neurotrophic factors is much more limited and comes from a single study. These findings support the biological plausibility that early systemic inflammation and altered neurotrophic signalling may identify preterm infants at increased risk for motor delay, while also highlighting substantial heterogeneity and important gaps in the current literature.

While systemic inflammatory mediators are not brain-specific and may be perceived as “nonspecific”, they plausibly capture an early inflammatory burden that can trigger or amplify neuroinflammatory cascades during windows of heightened white-matter vulnerability in prematurity. From a translational perspective, their potential value lies in complementing—rather than replacing—clinical risk assessment and neuroimaging within prognostic models, helping to prioritise neurodevelopmental follow-up among high-risk preterm infants. However, the current evidence remains insufficient to support stand-alone screening or routine clinical decision-making, given heterogeneity in assays, sampling windows, neonatal morbidities and outcome instruments, as well as the absence of validated reference ranges and clinically applicable cut-offs. In future prognostic work, biomarker-based models should be evaluated for incremental value when added to existing NICU risk scores and clinical risk stratification tools (e.g., gestational age, major neonatal morbidities, and neuroimaging findings), rather than as isolated predictors. Mechanistically, systemic inflammatory surges occurring during developmental windows of heightened white-matter vulnerability in prematurity may plausibly amplify microglial-mediated injury pathways and disrupt oligodendrocyte maturation, providing a rationale for time-sensitive sampling strategies in longitudinal cohorts. This perspective supports biomarkers as complementary tools to refine risk communication and prioritise follow-up, while recognising that current heterogeneity precludes clinically applicable thresholds.

Among the eight cohorts included, three were rated as high methodological quality and five as moderate quality according to the Newcastle–Ottawa Scale (NOS), mostly due to limited adjustment for confounding variables and incomplete reporting of follow-up. These limitations reduce confidence in the magnitude and consistency of the observed associations and indicate that the findings should be interpreted as hypothesis-generating rather than confirmatory. Despite these weaknesses at the level of the primary studies, the present systematic review was rated as high according to the AMSTAR-2 appraisal, with no critical weaknesses and a single non-critical weakness related to the lack of systematic extraction of funding sources from the primary studies. This supports the robustness and transparency of the review methods and reporting, even though the underlying evidence remains observational and prone to residual confounding. However, the AMSTAR-2 appraisal was conducted by members of the author team (self-appraisal), and some degree of subjectivity cannot be excluded; therefore, the review-level rating may be optimistic and should be interpreted with caution.

Most of the included cohorts enrolled very preterm or extremely preterm infants and reported a high burden of neonatal morbidities, such as late-onset sepsis, necrotising enterocolitis, chronic lung disease, severe intraventricular haemorrhage, periventricular leukomalacia and retinopathy of prematurity [13,14,15,16,17,30]. In large prospective cohorts, preterm neonates who exhibited persistently elevated levels of inflammatory proteins during the first two postnatal weeks were more likely to present severe developmental limitations at 24 months of age [14]. In other studies, higher concentrations of IL-6, IL-8, TNF-α and CRP during the neonatal period were associated with lower scores on motor scales at follow-up [6,13,14,15,16,17,30]. These results are consistent with experimental and clinical data suggesting that systemic inflammation during critical windows of brain development may contribute to white matter injury, altered synaptic organisation and long-term motor impairment [6,7,23]. At the same time, null findings in some analyses, particularly in the ELGAN-related cohorts [12,30], underscore that these associations are not universal and may depend on the timing, intensity and chronicity of the inflammatory response, as well as on the clinical context in which biomarkers are measured.

Several factors may explain the divergent results across studies. First, gestational age and birth weight distributions varied substantially, and extremely preterm infants are intrinsically at higher risk of motor impairment, regardless of inflammatory biomarker levels [7,12]. Second, the burden and profile of neonatal morbidities differed between cohorts, with some studies including infants with sepsis and other acute inflammatory conditions and others excluding such cases [6,13,14,15,16,17,30]. Third, there was marked heterogeneity in biospecimens (blood versus urine), sampling windows (from cord blood at birth to repeated measurements over the first two postnatal weeks or until 35 weeks of postmenstrual age) and laboratory methods (MSD multiplex, ELISA, electrochemiluminescence). These differences affect the absolute concentrations measured and may influence the strength and direction of associations. For example, elevations in IL-1β and IL-8 measured in urine during the first days of life were associated with typical motor development in one study [16], in contrast to other cohorts in which higher cytokine levels in blood predicted worse outcomes [14,15,31]. Finally, motor outcomes were assessed using different instruments (BSID-II, BSID-III, TIMP, NAPI), at slightly different ages and with variable cut-offs, which further limits direct comparability. In addition to biological factors, demographic and contextual determinants such as maternal age, socio-economic status, access to specialised perinatal care and early intervention programmes may also influence both the risk of preterm birth and subsequent motor outcomes and were only partially captured in the available cohorts [5,6,7,8].

The available evidence on neurotrophic factors in preterm infants is even more limited. Magalhães et al. [16] reported that higher BDNF and GDNF concentrations were associated with poorer motor prognosis among infants with below-expected motor development, suggesting a possible compensatory upregulation in response to central nervous system injury. In contrast, other studies in broader neonatal populations have proposed that lower BDNF levels may serve as early markers of neurodevelopmental impairment, particularly in the motor domain, and that maternal lifestyle and perinatal exposures can modulate BDNF levels [8,22]. Taken together, these findings indicate that neurotrophic factors are biologically plausible candidates for risk stratification but cannot yet be considered established predictors of motor outcomes in preterm infants.

It is important to emphasise that all associations reported in this review derive from observational cohort studies and therefore do not allow causal inferences. Elevated inflammatory biomarkers and altered neurotrophic factor levels may reflect upstream processes related to prematurity, infection, hypoxia–ischaemia or other complications, rather than being direct causes of motor impairment. Residual confounding by factors such as socio-economic status, perinatal care, nutritional status and the quality of early intervention cannot be excluded. Moreover, most cohorts were conducted in tertiary neonatal intensive care units in high-resource settings, and primarily included infants at the extremes of prematurity. These characteristics limit the generalisability of the findings to preterm populations with different risk profiles, to settings with fewer resources and to longer-term outcomes beyond 24 months.

This review has several strengths, including prospective registration in PROSPERO, adherence to PRISMA and Cochrane guidance, duplicate study selection and data extraction, and formal quality assessment of both the primary cohorts (using the NOS) and the review itself (using AMSTAR-2). At the same time, important limitations need to be acknowledged. To the best of our knowledge, there are no established reference ranges for inflammatory biomarkers in preterm neonates that can be used to define risk thresholds for motor delay. The heterogeneity in study designs, biomarker panels, laboratory methods and motor assessment tools, as well as the modest sample sizes of some cohorts, precluded meta-analysis and quantitative estimation of effect sizes. Part of this methodological variability likely reflects temporal changes in neonatal care practices, neurodevelopmental follow-up protocols and biomarker assay technologies over the 2011–2023 period covered by the included cohorts. Moreover, the available cohorts rarely reported longitudinal time-course trajectories linking serial biomarker changes to repeated clinical neurological scoring and repeated motor “points” across development, which precluded a dynamic synthesis of neuroplasticity/neurodestruction markers over time. In addition, the biomarker scope of this review was restricted a priori to inflammatory mediators/acute-phase proteins and neurotrophic/astroglial biomarkers as defined in our search strategy, and therefore did not specifically target other mechanistic biomarker domains relevant to neonatal brain injury—such as oxidative/nitrosative stress, energy metabolism/mitochondrial dysfunction, endothelial dysregulation, or cellular stress-response proteins (e.g., HSP70). These complementary domains are provided as contextual background and future research directions in Supplementary Table S5, and should be evaluated in targeted systematic reviews and larger cohorts to determine whether multimodal panels improve prognostic performance when combined with key neonatal morbidities and clinical risk factors.

Nevertheless, the present review provides a comprehensive and methodologically robust synthesis of the available evidence, identifies key sources of heterogeneity and highlights priorities for future research. This review adds value by providing an updated synthesis focused specifically on motor outcomes up to 24 months in preterm cohorts, while detailing key sources of methodological heterogeneity (biospecimens, sampling windows, assay platforms, neonatal morbidities, and outcome instruments) that currently limit quantitative pooling and clinical translation. In addition, NICU length of stay (LOS) was inconsistently reported across cohorts, limiting comparability and potentially confounding biomarker–motor outcome associations, as LOS may partially capture illness severity and cumulative exposure to inflammatory drivers (e.g., infection burden and duration of ventilatory support). From a practical standpoint, early elevations in IL-6, IL-8, TNF-α and CRP emerge as the most consistently reported signals among the assessed biomarkers; although they are not CNS-specific, they may be interpreted as indicators of systemic inflammatory burden and can support risk communication and prioritisation of neurodevelopmental follow-up when these biomarkers are already collected for clinical reasons.

However, evidence remains insufficient for stand-alone screening or for routine biomarker-based monitoring of neuroprotective therapy, given the absence of validated reference ranges, cut-offs and standardised longitudinal protocols. Moreover, assay-related measurement variability across platforms may introduce differential measurement error and reduce comparability of biomarker levels across cohorts, contributing to heterogeneity and limiting quantitative synthesis and translation into clinically applicable thresholds. Future prognostic research should evaluate whether multimodal panels combining systemic inflammatory markers with CNS/brain-injury biomarkers (e.g., GFAP, S100B, NSE, UCH-L1, NfL) improve prognostic performance for movement-related outcomes in preterm infants (Supplementary Table S5). As contextual background and future research directions, Supplementary Table S5 also summarises representative biomarkers within each mechanistic domain and their potential prognostic and mechanistic rationale in neonatology-related settings, supported by key references. Larger, multicentre cohorts with standardised biomarker panels, harmonised sampling protocols, detailed characterisation of neonatal morbidities and long-term follow-up using validated motor scales are needed to clarify the prognostic value of inflammatory and neurotrophic/astroglial biomarkers and to determine whether they can be incorporated into clinically useful risk stratification tools for preterm infants.

## 5. Conclusions

The findings of this systematic review suggest that elevated neonatal levels of inflammatory biomarkers, particularly IL-6, IL-8, TNF-α and C-reactive protein, are often associated with less favourable motor or neuropsychomotor development in preterm NB up to 24 months of corrected age, whereas evidence on neurotrophic factors is limited to a single cohort. Accordingly, neurotrophic biomarker findings should be considered preliminary and cannot currently inform clinical screening or stand-alone prognostic prediction in preterm infants. Taken together, the available data support the hypothesis that early systemic inflammation and altered neurotrophic signalling may contribute to increased risk of motor delay in this population, but they are not yet sufficient to establish causality or to define clinically applicable cut-off values. Future large, methodologically rigorous cohorts using standardised biomarker panels and harmonised motor assessment tools are needed to confirm these associations and to clarify the role of inflammatory and neurotrophic biomarkers in routine prognostic assessment of preterm infants.

## Figures and Tables

**Table 1:** Assessment of cohort study quality according to the Newcastle–Ottawa Scale (NOS).

Author/Year	Representativeness of Exposed Cohort	Selection of Non-Exposed Cohort	Ascertainment of Exposure	Outcome Not Present at Start	Comparability of Cohorts (Design/Analysis)	Assessment of Outcome	Follow-Up Long Enough for Outcomes	Adequacy of Follow-Up	Total Score (0–9)	Overall Quality
Silveira et al. [15]	–	+	–	+	++	–	+	–	5/9	Moderate
O’Shea et al. [14]	+	+	+	+	+–	–	+	–	6/9	Moderate
Rose et al. [13]	–	+	–	+	++	+	+	+	7/9	High
Kuban et al. [12]	+	+	–	+	++	–	+	+	7/9	High
Magalhães et al. [16]	–	–	–	+	++	+	+	+	6/9	Moderate
Nist et al. [31]	–	–	+	+	++	–	–	–	4/9	Moderate
Lee et al. [17]	–	+	–	+	++	–	+	+	6/9	Moderate
Kurul et al. [30]	–	+	+	+	+–	+	+	+	7/9	High

A cross (+) indicates that the study fulfilled the NOS criterion and a dash (–) that the criterion was not met. In the comparability domain, up to two stars (represented by “++”) can be awarded. The NOS total score ranges from 0 to 9. We considered studies with ≥7 stars as high quality, 4–6 stars as moderate quality, and <4 stars as low quality.

**Table 2:** Characteristics of studies that evaluated inflammatory and/or neurotrophic biomarkers in preterm neonates as predictive factors for neuropsychomotor development.

Author/Year	Patients (*n*)	Gestational Age at Birth (Weeks)	Birth Weight (g)	Key Neonatal Morbidities *	NICU Length of Stay (Days)	Analysed Material	Laboratory Technique	Inflammatory and Neurodevelopmental Markers	Neuropsychomotor Development Assessment Scale
Silveira et al. [15]	62	29.5 ± 2	1100 ± 220	Respiratory distress syndrome, *n* = 33; Positive blood culture 72 h, *n* = 5; Periventricular leukomalacia, *n* = 16; Intraventricular haemorrhage (grades III/IV), *n* = 10; Bronchopulmonary dysplasia, *n* = 6.	Not reported	Blood	Human cytokine elenco plex kit	IL-6, IL-1β, IL-8, IL-10 and TNF-α	BSID-II
O’Shea et al. [14]	939	≤28	-	Chronic lung disease, *n* = 52; Late onset sepsis, *n* = 26; Necrotizing enterocolitis, *n* = 12; Moderate/severe IVH, *n* = 21; Ventricular enlargement, *n* = 10.	Not reported	Blood	MSD Multiplex	IL-1β, IL-6, TNF-α, IL-8, CRP	BSID-II e GMFCS
Rose et al. [13]	102	28.7 ± 2.4	1087 ± 279	Necrotizing enterocolitis, *n* = 12; Retinopathy of prematurity, *n* = 28. Bronchopulmonary dysplasia, *n* = 27; Sepsis, *n* = 10.	Not reported	Blood	-	CRP	BSID-III
Kuban et al. [12]	881	≤28	-	Not reported	Not reported	Blood	MSD Multiplex	IL-1 β, IL-6, and TNF-α	BSID-II
Magalhães et al. [16]	40	30 ± 1	1477 ± 428	Neonatal morbidities not reported	Not reported	Blood and Urine	MSD Multiplex	IL-1β, IL-6, IL-10, TNF-α, BDNF, GDNF	TIMP
Nist et al. [31]	68	30 ± 1.1	770 ± 233	Associated inflammatory conditions explicitly excluded (see Methods); other neonatal morbidities not reported	Not reported	Blood	MSD Multiplex	IL-6, IL-8, IL-1β, TNF-α and IL-10	NAPI
Lee et al. [17]	45	24–33	420–1400	Respiratory distress syndrome, *n* = 35; Bronchopulmonary dysplasia, *n* = 30; Retinopathy of prematurity, *n* = 15; Intraventricular haemorrhage (grades III), *n* = 2; Necrotizing enterocolitis (stage II), *n* = 2; Sepsis, *n* = 11	Not reported	Blood	ELISA	IL-1β, IL-6, IL-8 and TNF-α	BSID-III
Kurul et al. [30]	326	≤30	410–2000	Sepsis, 1 episode, *n* = 86; Sepsis, with more than 1 episode, *n* = 50	Not reported	Blood	Cobas^®^ 8000 System, Roche Diagnostics	IL-6 and CRP	Bayley-III-NL

*: Number of episodes; Mean ± SD; Minimum–maximum; ≥: greater than or equal to; ≤: less than or equal to; MSD—Meso Scale Discovery electrochemiluminescence system; IL-6: interleukin-6; IL-8: interleukin 8; IL-1β: interleukin-1 beta; IL-10: interleukin 10; TNF-α: tumour necrosis factor alpha; CRP: C-reactive protein; BDNF: Brain-derived neurotrophic factor; GDNF: Glial Cell Line-derived Neurotrophic Factor; BSID II: Bayley Scales of Infant Development, Second Edition; BSID III: Bayley Scales of Infant Development, Third Edition; GMFCS: Gross Motor Function Classification System; TIMP: Test of Infant Motor Performance; NAPI: Neurobehavioral Assessment of the Preterm Infant; Bayley-III-NL: Dutch Bayley Scales of Infant and Toddler Development, third edition.

**Table 3:** Levels of inflammatory and/or neurotrophic biomarkers in preterm neonates as predictors of motor development.

Author/Year	Biological Material Collection Time(s)	Development Assessment Time	Conditions That Can Generate Inflammatory Processes *	Inflammatory and/or Neurodevelopmental Biomarker	Motor Delay (Scales Used)	Main Results
Silveira et al. [15]	-	22–24 months CA	Present	IL-6, IL-8, IL-10, IL-1β and TNF-α	BSID-II, an PDI > 84 is defined as normal	Regarding PDI, high levels of IL-6, IL-8 and TNF-α, present an increased risk of PDI < 85
O’Shea et al. [14]	Drops of blood were collected on filter paper, 1 postnatal day (break, day 1–3), on the seventh day (break, day 5–8) and day 14 (break, day 12–15)	24 months CA	Present	IL-1β, IL-6, TNF-α, IL-8 and CRP	Developmental assessments included the BSID-II (score < 55) and an assessment of gross motor function using the GMFCS	Regarding motor development, delayed babies (score < 55) showed increased levels of the inflammatory biomarker CRP in the 7th day sample and of the inflammatory biomarkers IL-1b, IL-6, TNF-a, IL-8 and CRP on the 14th day
Rose et al. [13]	First two weeks of life	18–22 months CA	Present	CRP	BSDI III with motor score < 85	The inflammation biomarker CRP was significantly higher in children with motor scores < 85. Furthermore, the higher mean CRP correlates with lower motor and fine motor scores (<85)
Kuban et al. [12]	Blood samples collected on the first, seventh and fourteenth postnatal days	24 months CA	-	IL-1, IL-6, IL-6R, TNF-α	Developmental assessments included the BSID-II (PDI < 55)	There was no significant association between the values obtained in the PDI and the concentrations of inflammatory biomarkers at two years of age
Magalhães et al. [16]	Umbilical cord blood (T0), at 48 (T1) and 72 h (T2) of life and finally at 3 weeks after birth (T3). Urine samples were obtained at the same time points as peripheral blood collection after birth	34 weeks GA	Present	L-1β, IL-6, IL-10, TNF-α, BDNF and GDNF	The TIMP was applied to assess motor development, with babies classified into two groups: “typical motor development” (values equal to or greater than the 5th percentile) and “below expected” (values below the 5th percentile)	Higher IL-1β values were found in the group with typical motor development using TIMP. With urine, a significant increase in BDNF and GDNF was found in the group with motor development below expected at T2 and T3, respectively
Nist et al. [31]	A blood sample was collected from the first week to 35 weeks of postnatal life, weekly	35 weeks GA	Absent	IL-6, IL-8, IL-1β, and TNF-α	NAPI scores in the MDV cluster are based on performance on 7 unique items	Babies with higher levels of TNF-α had lower NAPI scores in the MDV cluster. By including IL-6, TNF-α and IL-8 observed that higher composite inflammation scores were associated with decreased NAPI and MDV scores
Lee et al. [17]	Blood samples were collected on the first day of symptoms of systemic inflammation, two and six days later	18 months CA	Present	IL-1β, IL-6, and TNF-α	The BSDI III scale was used but they did not mention the score used	Among the biomarkers studied, TNF-α showed a negative and significant correlation in the motor domain of BSDI III
Kurul et al. [30]	Blood collection was carried out when the baby showed two or more symptoms of sepsis within 72 h of birth	24 months CA	Present	IL-6, and CRP	Bayley-III-NL, NDI serious < 70 e NDI light > 70	Regarding motor development, the increase in CRP concentration associated with sepesis was significantly associated with a motor score < 70. And a higher risk of severe NDI. Not observed for IL-6

CA: Correct Age; GA: gestational age; IL-6: interleukin-6; IL-8: interleukin 8; IL-1β: interleukin-1 beta; IL-10: interleukin 10; TNF-α: tumour necrosis factor alpha; IL-6R: receptor de interleukin 6; CRP: C-reactive protein; BDNF: Brain-derived neurotrophic factor; GDNF: Glial Cell Line-derived Neurotrophic Factor; BSID II: Bayley Scales of Infant Development, Second Edition; BSID III: Bayley Scales of Infant Development, Third Edition; GMFCS: Gross Motor Function Classification System; TIMP: Test of Infant Motor Performance; NAPI: Neurobehavioral Assessment of the Preterm Infant. MDV: Motor Development and igour; PDI: Psychomotor Developmental Index; Bayley-III-NL: Dutch Bayley Scales of Infant and Toddler Development, third edition; NDI: neurodevelopmental impairment. * Late-onset sepsis and/or necrotizing enterocolitis and/or chronic lung disease and/or ventricular enlargement and/or parenchymal echolucent lesion on cranial ultrasound and/or acute disorders and/or death within the first three weeks of life.

## Data Availability

The data used in this systematic review were extracted from previously published studies, which are publicly accessible through their respective journals and databases. All data generated or analysed during this study are included in this published article and its Supplementary Files.

## Supplementary Materials

The following supporting information can be downloaded at: https://www.mdpi.com/article/10.3390/pediatric18010007/s1, Table S1: PRISMA 2020 checklist for this systematic review; Table S2: Full electronic search strategies and applied filters for all databases; Table S3: Full-text articles excluded after eligibility assessment and main reasons for exclusion [19,32,33,34,35,36,37,38,39,40,41,42,43,44,45,46,47,48,49,50,51,52]; Table S4: Methodological quality assessment of the present systematic review according to the AMSTAR-2 tool; Table S5: Candidate biomarker domains relevant to neonatal brain injury and potential clinical utility (contextual background and future research directions) [24,25,53,54,55,56,57,58,59,60,61,62,63,64,65,66,67,68,69].

## Abbreviations

NBs	newborns
NOS	Newcastle-Ottawa Scale
WHO	World Health Organization
PRISMA	Preferred Reporting Items for Systematic Reviews and Meta-Analyses
IL-1β	interleukin-1 beta
IL-6	interleukin-6
IL-8	interleukin-8
IL-10	interleukin-10
CRP	C-reactive protein
TNF-α	tumour necrosis factor alpha
GFAP	glial fibrillary acidic protein
GDNF	glial cell-derived neurotrophic factor
BSID II	Bayley Scales of Infant Development, Second Edition
BSID III	Bayley Scales of Infant Development, Third Edition
TIMP	Test of Infant Motor Performance
NAPI	Neurobehavioral Assessment of the Preterm Infant
PDI	Psychomotor Development Index
MDV	Motor Development and Vigour
NDI	Neurodevelopmental impairment
MD	Motor development
