# Supplementary material for: Inflammatory Biomarkers and Neurotrophic Factors in Preterm Newborns as Predictors of Motor Development: A Systematic Review
Source: Pediatr Rep. 2026 Jan 5;18(1):7. doi: 10.3390/pediatric18010007 (PMC12821560; doi:10.3390/pediatric18010007)
Supplement: Supplementary file 1 [file pediatrrep-18-00007-s001.zip › pediatrrep-4036088-supplementary/pediatrrep-4036088-supplementary.pdf]

Supplementary Table S1 – PRISMA 2020 Checklist

**Section and topic**	**Item #**	**Checklist item**	**Location where item is reported**
TITLE	1	Identify the report as a systematic review.	Title page (title: "Inflammatory biomarkers and neurotrophic factors in preterm neonates as predictors of motor development: a systematic review")
ABSTRACT	2	Provide a structured summary of the review.	Abstract (Background/Objectives, Methods, Results, Conclusions)
INTRODUCTION	3	Rationale: describe the rationale for the review.	Introduction, paragraphs 1–5 (context of prematurity, NPMD, biomarkers, inflammation/neurotrophins)
INTRODUCTION	4	Objectives: provide an explicit statement of the objectives or questions.	Introduction, final paragraph (PCC structure and explicit objective)
METHODS	5	Eligibility criteria: specify inclusion and exclusion criteria and how studies were grouped.	Methods, section 2.1 (eligibility criteria and exclusion reasons); Results, section 3.1; Supplementary Table S3 (list of excluded full texts and reasons)
METHODS	6	Information sources: specify all databases and other sources, with dates.	Methods, section 2.1 (databases, search dates including July 2024 update)
METHODS	7	Search strategy: present full search strategies for all databases.	Methods, section 2.1; Supplementary Table S2 (complete, copy-pasteable search strings and filters)
METHODS	8	Selection process: specify methods used to select studies, including how many reviewers, and how disagreements were resolved.	Methods, section 2.1 (screening in Covidence, three independent reviewers, fourth reviewer for disagreements); Figure 1 (PRISMA flow diagram)
METHODS	9	Data collection process: specify methods used to collect data (how many reviewers, whether piloted forms were used, etc.).	Methods, section 2.2 (data extraction by three reviewers and resolution of discrepancies)
METHODS	10a	Data items (outcomes): list and define all outcomes sought, and how multiple measures were handled.	Methods, section 2.2 (NPMD scales, motor outcomes, timing of assessment); Results, sections 3.4–3.7; Tables 2–3

METHODS	10b	Data items (other variables): list and define other variables (e.g., participant and intervention characteristics).	Methods, section 2.2 (gestational age, birth weight, biospecimen type, collection times, laboratory methods, neonatal morbidities); Results, section 3.4; Table 2
METHODS	11	Study risk of bias assessment: specify methods used to assess risk of bias in included studies.	Methods, section 2.2 (Newcastle–Ottawa Scale description, domains, scoring and reviewers); Results, section 3.2; Table 1
METHODS	12	Effect measures: specify for each outcome the effect measure used.	Not applicable (no meta-analysis or pooled effect measures were calculated)
METHODS	13a	Synthesis methods: describe process for deciding which studies were eligible for each synthesis.	Methods, section 2.4 (Data analysis – justification for narrative synthesis only); Results, sections 3.4–3.7
METHODS	13b	Synthesis methods: methods required to prepare data for synthesis (handling missing data, transformations, etc.).	Not applicable (no statistical synthesis or data transformation was performed)
METHODS	13c	Synthesis methods: methods used to tabulate or visually display results.	Methods, section 2.4 (description of narrative and tabular synthesis); Results, sections 3.4–3.7; Tables 1–3
METHODS	13d	Synthesis methods: methods used to synthesize results and rationale (e.g., meta-analysis model).	Not applicable (no meta-analysis or statistical synthesis was performed)
METHODS	13e	Synthesis methods: methods used to explore possible causes of heterogeneity.	Not applicable (no quantitative exploration of heterogeneity; heterogeneity is described qualitatively in Discussion)
METHODS	13f	Synthesis methods: sensitivity analyses conducted.	Not applicable (no sensitivity analyses were performed)
METHODS	14	Reporting bias assessment: methods used to assess risk of bias due to missing results (reporting bias).	Not applicable (no meta-analysis or formal assessment of reporting bias was performed)
METHODS	15	Certainty assessment: methods used to assess certainty (e.g., GRADE).	Not applicable (no formal certainty assessment such as GRADE was performed)

RESULTS	16a	Study selection: describe search results and how many records were screened, assessed, and included.	Results, section 3.1 (numbers at each stage); Figure 1 (PRISMA flow diagram)
RESULTS	16b	Study selection: cite studies that appeared to meet criteria but were excluded, and explain why.	Results, section 3.1; Supplementary Table S3 (list of 22 excluded full texts with reasons)
RESULTS	17	Study characteristics: present characteristics of included studies.	Results, section 3.4 (cohort characteristics); Table 2 (sample sizes, GA, birth weight, morbidities, NICU stay, biomarkers, scales)
RESULTS	18	Risk of bias in studies: present risk of bias assessments.	Results, section 3.2; Table 1 (NOS scores and classification of methodological quality)
RESULTS	19	Results of individual studies: present summary statistics and effect estimates for each study.	Results, section 3.7 (associations between biomarkers and outcomes); Table 3 (main findings for each cohort)
RESULTS	20a	Results of syntheses: summarise characteristics and risk of bias among contributing studies.	Not applicable (no quantitative synthesis); narrative synthesis and risk of bias are presented in Results (3.2–3.7) and discussed in Discussion (section 4)
RESULTS	20b	Results of syntheses: present results of all statistical syntheses (including heterogeneity).	Not applicable (no meta-analysis or other statistical synthesis)
RESULTS	20c	Results of syntheses: present results of investigations of heterogeneity.	Not applicable (no statistical investigation of heterogeneity; heterogeneity described qualitatively in Discussion, section 4)
RESULTS	20d	Results of syntheses: present results of sensitivity analyses.	Not applicable (no sensitivity analyses were conducted)
RESULTS	21	Reporting biases: present assessment of risk of bias due to missing results.	Not applicable (no formal assessment of reporting bias; see Methods, sections 2.3–2.4)
RESULTS	22	Certainty of evidence: present assessment of certainty for each outcome.	Not applicable (no formal certainty assessment such as GRADE was performed)

DISCUSSION	23a	Provide a general interpretation of the results in the context of other evidence.	Discussion, section 4, first and third paragraphs (summary of associations; comparison with experimental and clinical data and external studies)
DISCUSSION	23b	Discuss limitations of the included evidence.	Discussion, section 4 (paragraphs 2–5: observational design, sample size, heterogeneity, limited data on neurotrophic factors, incomplete confounder control)
DISCUSSION	23c	Discuss limitations of the review processes used.	Discussion, section 4, penultimate paragraph (heterogeneity, lack of reference ranges, impossibility of meta-analysis, temporal changes across 2011–2023)
DISCUSSION	23d	Discuss implications of the results for practice, policy, and future research.	Discussion, last paragraph; Conclusions, section 5 (need for larger multicentre cohorts, standardisation, implications for future prognostic tools)
OTHER INFORMATION	24a	Registration: provide registration information.	Abstract (PROSPERO CRD42022365839); Methods, section 2 (PROSPERO registration)
OTHER INFORMATION	24b	Protocol: indicate where the protocol can be accessed.	Methods, section 2 (reference to PROSPERO registration and protocol)
OTHER INFORMATION	24c	Amendments: describe and explain any amendments to information provided at registration or in the protocol.	Not applicable (no major deviations from the registered protocol)
OTHER INFORMATION	25	Support: describe sources of financial or non-financial support and the role of funders.	Funding/Support statement (end of manuscript)
OTHER INFORMATION	26	Competing interests: declare any competing interests of review authors.	Conflicts of Interest statement (end of manuscript)
OTHER INFORMATION	27	Availability of data, code and other materials.	Data Availability Statement; Supplementary Materials (Tables S1–S4 and additional files)

Supplementary Table S2 – Detailed database search strategies

**Databases:**	**Search strategy:**	**Filters:**
**Pubmed:**	("Child Development"[Mesh] OR "Child Development Disorders, Pervasive"[Mesh] OR "Growth and Development"[Mesh] OR "Developmental Disabilities"[Mesh] OR "child development"[tiab] OR "infant development"[tiab] OR "growth and development"[tiab] OR "developmental delay*"[tiab] OR "motor development"[tiab] OR "psychomotor development"[tiab]) AND ("Child"[Mesh] OR "Infant"[Mesh] OR "Infant, Newborn"[Mesh] OR infant*[tiab] OR newborn*[tiab] OR baby*[tiab]) AND ("Environment"[Mesh] OR "Environmental Impact*"[tiab] OR "Home Environment"[Mesh] OR "Socioeconomic Factors"[Mesh] OR "Risk Factor*"[Mesh] OR "Environmental Exposure*"[tiab] OR "Social Determinants of Health"[tiab] OR "socioeconomic status"[tiab] OR "housing conditions"[tiab] OR "parental education"[tiab] OR "family environment"[tiab]) AND "Observational Study"[Publication Type]	Observational study, cohort analysis, cross-sectional study, case control study, Humans, Female, Male, Newborn: birth-1 month, Infant: birth-23 months.
**Embase:**	("premature" OR "premature AND birth" OR "newborn" OR preterm" **AND** "cytokine" OR "inflammation" OR "'il 6'" OR "'il 1Î^{2}'" OR "'il 8'" OR "'l 10'" OR "'tnf alfa'" OR "'glial cell line derived neurotrophic factor'" OR "gdnf" OR "'nerve growth factor'" OR "'neurotrophic factors'" OR "bdnf" OR "'brain derived neurotrophic factor'" OR "'gfap protein'" OR "biomarkers" **AND** "'growth neurodevelopment'" OR "'motor development'" OR "'neuropsychomotor development'")	Observational study, cohort analysis, cross-sectional study, case control study, Humans, Female, Male, Newborn: birth-1 month, Infant: birth-23 months.
**Web of Sciencie:**	TS=((premature OR "premature birth" OR newborn* OR preterm) AND (cytokine OR inflammation OR "IL-6" OR "IL 6" OR "interleukin 6" OR "IL-1β" OR "IL 1β" OR "interleukin 1 beta" OR "IL-8" OR "IL 8" OR "interleukin 8" OR "IL-10" OR "IL 10" OR "interleukin 10" OR "TNF alfa" OR "TNF alpha" OR "tumor necrosis factor alpha" OR GDNF OR "glial cell line derived neurotrophic factor" OR NGF OR "nerve growth factor" OR BDNF OR "brain derived neurotrophic factor" OR "GFAP protein" OR "glial fibrillary acidic protein" OR biomarker*) AND ("growth neurodevelopment" OR "motor development" OR "neuropsychomotor development"))	Not applicable.

**Scielo:**	(Premature OR Premature birth OR Newborn OR Preterm **AND** Cytokine OR Inflammation OR IL-6 OR IL-1β OR IL-8 OR L-10 OR TNF-alfa OR PCR OR Neurotrophic factors OR Nerve Growth Factors OR GDNF OR Glial Cell Line-Derived Neurotrophic Factors OR BDNF OR Brain-Derived Neurotrophic Factor OR GFAP protein OR Biomarkers **AND** Growth neurodevelopment OR Motor development OR Neuropsychomotor development)	Not applicable

Supplementary Table S3 – Full-text articles excluded after eligibility assessment and reasons for exclusion

Ref. no.	First author, year	Title	Main reason for exclusion
1	Bartha A.I., 2004 [32]	Neonatal encephalopathy: association of cytokines with MR spectroscopy and outcome.	Study design outside the inclusion criteria.
2	Bayman M.G., 2022 [33]	Foetal umbilical cord brain-derived neurotrophic factor (BDNF) levels in pregnancy with gestational diabetes mellitus.	Study design outside the inclusion criteria.
3	Caesar R., 2016 [34]	Early prediction of typical outcome and mild developmental delay for prioritisation of service delivery for very preterm and very low birthweight infants: a study protocol.	Study design outside the inclusion criteria (protocol study).
4	Clark E.A., 2011 [35]	Repeated course antenatal steroids, inflammation gene polymorphisms, and neurodevelopmental outcomes at age 2.	Population/outcomes outside the scope of the review (no neonatal biomarker–motor outcome relationship in preterm infants).
5	Cooper D.M., 2020 [36]	Body composition and neuromotor development in the year after NICU discharge in premature infants.	Study design outside the inclusion criteria (no neonatal inflammatory/neurotrophic biomarker assessment).
6	Dewi M., 2017 [37]	Programming of infant neurodevelopment by maternal obesity: potential role of maternal inflammation and insulin resistance.	Population/outcomes outside the scope (focus on maternal factors, not neonatal biomarkers in preterm infants).

7	Dietrick B., 2020 [38]	Plasma and cerebrospinal fluid candidate biomarkers of neonatal encephalopathy severity and neurodevelopmental outcomes.	Population outside the scope (neonatal encephalopathy, not restricted to preterm infants with motor outcomes).
8	Harding D., 2005 [39]	Variation in the interleukin-6 gene is associated with impaired cognitive development in children born prematurely: a preliminary study.	Study design outside the inclusion criteria (genetic polymorphism study; outcomes not limited to motor development).
9	Harding D.R., 2004 [40]	Does interleukin-6 genotype influence cerebral injury or developmental progress after preterm birth?	Absence of a validated neuropsychomotor development assessment scale focusing on motor outcomes.
10	Kapitanović V.H., 2012 [41]	The association between proinflammatory cytokine polymorphisms and cerebral palsy in very preterm infants.	Absence of a validated neuropsychomotor development assessment scale as defined in the eligibility criteria.
11	Kohman R.A., 2008 [42]	Neonatal endotoxin exposure impairs avoidance learning and attenuates endotoxin-induced sickness behavior and central IL-1β gene transcription in adulthood.	Animal model; no human preterm neonatal population with motor scale assessment.
12	Pascual-Mancho J., 2022 [43]	Brain-derived neurotrophic factor levels in cord blood from growth restricted fetuses with Doppler alteration compared to adequate for gestational age fetuses.	Absence of a validated neuropsychomotor development assessment scale up to 24 months.
13	Ragsdale H.B., 2019 [19]	Regulation of inflammation during gestation and birth outcomes: Inflammatory cytokine balance predicts birth weight and length.	Population/outcomes outside the scope (birth anthropometrics, without motor development assessment).

14	Ren L.H., 2016 [44]	Prenatal lead exposure related to cord blood brain derived neurotrophic factor (BDNF) levels and impaired neonatal neurobehavioral development.	Absence of a validated neuropsychomotor development scale focusing on motor outcomes up to 24 months.
15	Uguz F., 2013 [45]	Maternal generalized anxiety disorder during pregnancy and fetal brain development: a comparative study on cord blood brain-derived neurotrophic factor levels.	Topic outside the scope of the review (maternal psychiatric disorder; no longitudinal motor development assessment in preterm infants).
16	Voltas N., 2017 [46]	Are there early inflammatory biomarkers that affect neurodevelopment in infancy?	Population/outcomes outside the scope (not restricted to preterm infants and motor development as defined in the review).
17	Aisa M.C., 2021 [47]	Urinary Nerve Growth Factor in full-term, preterm and intra uterine growth restriction neonates: Association with brain growth at 30–40 days of postnatal period and with neuro-development outcome at two years. A pilot study.	Study design outside the inclusion criteria (mixed population; outcomes not limited to motor scales in preterm infants).
18	Nist M.D., 2020 [48]	Inflammatory predictors of neurobehavior in very preterm infants.	Duplicate data relative to an included cohort.
19	O'Shea T.M., 2013 [49]	Inflammation-initiating illnesses, inflammation-related proteins, and cognitive impairment in extremely preterm infants.	Absence of a validated motor or neuropsychomotor development scale as primary outcome.
20	Rao R., 2009 [50]	Brain-derived neurotrophic factor in infants <32 weeks gestational age: correlation with antenatal factors and postnatal outcomes.	Absence of a validated neuropsychomotor development assessment scale focusing on motor outcomes.

21	Wood T.R., 2021 [51]	Early biomarkers of hypoxia and inflammation and two-year neurodevelopmental outcomes in the preterm Erythropoietin Neuroprotection (PENUT) Trial.	Study design outside the inclusion criteria (randomised clinical trial with different primary aims).
22	Leviton A., 2013 [52]	Two-hit model of brain damage in the very preterm newborn: small for gestational age and postnatal systemic inflammation.	Absence of motor or neuropsychomotor development assessment using validated scales as defined in the eligibility criteria.

References (Table S3)

19. Ragsdale, H.B.; Kuzawa, C.W.; Borja, J.B.; Avila, J.L.; McDade, T.W. Regulation of inflammation during gestation and birth outcomes: Inflammatory cytokine balance predicts birth weight and length. *Am. J. Hum. Biol.* **2019**, *31*, e23245.
32. Bartha, A.I.; Foster-Barber, A.; Miller, S.P.; Vigneron, D.B.; Glidden, D.V.; Barkovich, A.J.; Ferriero, D.M. Neonatal encephalopathy: Association of cytokines with MR spectroscopy and outcome. *Pediatr. Res.* **2004**, *56*, 960–966.
33. Bayman, M.G.; Inal, Z.O.; Hayiroglu, F.; Ozturk, E.N.Y.; Gezginc, K. Foetal umbilical cord brain-derived neurotrophic factor (BDNF) levels in pregnancy with gestational diabetes mellitus. *J. Obstet. Gynaecol.* **2022**, *42*, 1097–1102.
34. Caesar, R.; Boyd, R.N.; Colditz, P.; Cioni, G.; Ware, R.S.; Salthouse, K.; Doherty, J.; Jackson, M.; Matthews, L.; Hurley, T.; et al. Early prediction of typical outcome and mild developmental delay for prioritisation of service delivery for very preterm and very low birthweight infants: A study protocol. *BMJ Open* **2016**, *6*, e010726.
35. Clark, E.A.; Mele, L.; Wapner, R.J.; Spong, C.Y.; Sorokin, Y.; Peaceman, A.; Iams, J.D.; Leveno, K.J.; Harper, M.; Caritis, S.N.; et al. Repeated course antenatal steroids, inflammation gene polymorphisms, and neurodevelopmental outcomes at age 2. *Am. J. Obstet. Gynecol.* **2011**, *205*, 79.e1–79.e5.
36. Cooper, D.M.; Girolami, G.L.; Kepes, B.; Stehli, A.; Lucas, C.T.; Haddad, F.; Zalidvar, F.; Dror, N.; Ahmad, I.; Soliman, A.; et al. Body composition and neuromotor development in the year after NICU discharge in premature infants. *Pediatr. Res.* **2020**, *88*, 459–465.
37. Dewi, M.; Carlson, S.E.; Gustafson, K.M.; Sullivan, D.K.; Wick, J.A.; Hull, H.R. Programming of infant neurodevelopment by maternal obesity: Potential role of maternal inflammation and insulin resistance. *Asia Pac. J. Clin. Nutr.* **2017**, *26*, S36–S39.
38. Dietrick, B.; Molloy, E.; Massaro, A.N.; Strickland, T.; Zhu, J.; Slevin, M.; Donoghue, V.; Sweetman, D.; Kelly, L.; O'Dea, M.; et al. Plasma and Cerebrospinal Fluid Candidate Biomarkers of Neonatal Encephalopathy Severity and Neurodevelopmental Outcomes. *J. Pediatr.* **2020**, *226*, 71–79.e5.
39. Harding, D.; Brull, D.; Humphries, S.E.; Whitelaw, A.; Montgomery, H.; Marlow, N. Variation in the interleukin-6 gene is associated with impaired cognitive development in children born prematurely: A preliminary study. *Pediatr. Res.* **2005**, *58*, 117–120.
40. Harding, D.R.; Dhamrait, S.; Whitelaw, A.; Humphries, S.E.; Marlow, N.; Montgomery, H.E. Does interleukin-6 genotype influence cerebral injury or developmental progress after preterm birth? *Pediatrics* **2004**, *114*, 941–947.
41. Kapitanović Vidak, H.; Catela Ivković, T.; Jokić, M.; Spaventi, R.; Kapitanović, S. The association between proinflammatory cytokine polymorphisms and cerebral palsy in very preterm infants. *Cytokine* **2012**, *58*, 57–64.

42. Kohman, R.A.; Tarr, A.J.; Sparkman, N.L.; Bogale, T.M.; Boehm, G.W. Neonatal endotoxin exposure impairs avoidance learning and attenuates endotoxin-induced sickness behavior and central IL-1beta gene transcription in adulthood. *Behav. Brain Res.* **2008**, *194*, 25–31.
43. Pascual-Mancho, J.; Pintado-Recarte, P.; Morales-Camino, J.C.; Romero-Román, C.; Hernández-Martin, C.; Bravo, C.; Bujan, J.; Alvarez-Mon, M.; Ortega, M.A.; De León-Luis, J. Brain-Derived Neurotrophic Factor Levels in Cord Blood from Growth Restricted Fetuses with Doppler Alteration Compared to Adequate for Gestational Age Fetuses. *Medicina* **2022**, *58*, 178.
44. Ren, L.H.; Mu, X.Y.; Chen, H.Y.; Yang, H.L.; Qi, W. Prenatal lead exposure related to cord blood brain derived neurotrophic factor (BDNF) levels and impaired neonatal neurobehavioral development. *Zhonghua Yu Fang Yi Xue Za Zhi* **2016**, *50*, 514–518.
45. Uguz, F.; Sonmez, E.O.; Sahingoz, M.; Gokmen, Z.; Basaran, M.; Gezginc, K.; Sonmez, G.; Kaya, N.; Erdem, S.S.; Cicekler, H.; et al. Maternal generalized anxiety disorder during pregnancy and fetal brain development: A comparative study on cord blood brain-derived neurotrophic factor levels. *J. Psychosom. Res.* **2013**, 75, 346–350.
46. Voltas, N.; Arija, V.; Hernández-Martínez, C.; Jiménez-Feijoo, R.; Ferré, N.; Canals, J. Are there early inflammatory biomarkers that affect neurodevelopment in infancy? *J. Neuroimmunol.* **2017**, *305*, 42–50.
47. Aisa, M.C.; Barbati, A.; Cappuccini, B.; De Rosa, F.; Gerli, S.; Clerici, G.; Kaptilnyy, V.A.; Ishenko, A.I.; Di Renzo, G.C. Urinary Nerve Growth Factor in full-term, preterm and intra uterine growth restriction neonates: Association with brain growth at 30–40 days of postnatal period and with neuro-development outcome at two years. A pilot study. *Neurosci Lett.* **2021**, *741*, 135459.
48. Nist, M.D.; Pickler, R.H.; Harrison, T.M.; Steward, D.K.; Shoben, A.B. Inflammatory predictors of neurobehavior in very preterm infants. *Early Hum. Dev.* **2020**, *147*, 105078.
49. O'Shea, T.M.; Shah, B.; Allred, E.N.; Fichorova, R.N.; Kuban, K.C.K.; Dammann, O.; Leviton, A.; ELGAN Study Investigators. Inflammation-initiating illnesses, inflammation-related proteins, and cognitive impairment in extremely preterm infants. *Brain Behav. Immun.* **2013**, *29*, 104–112.
50. Rao, R.; Mashburn, C.B.; Mao, J.; Wadhwa, N.; Smith, G.M.; Desai, N.S. Brain-derived neurotrophic factor in infants < 32 weeks gestational age: Correlation with antenatal factors and postnatal outcomes. *Pediatr. Res.* **2009**, *65*, 548–552.
51. Wood, T.R.; Parikh, P.; Comstock, B.A.; Law, J.B.; Bammler, T.K.; Kuban, K.C.; Mayock, D.E.; Heagerty, P.J.; Juul, S.; PENUT Trial Consortium. Early Biomarkers of Hypoxia and Inflammation and Two-Year Neurodevelopmental Outcomes in the Preterm Erythropoietin Neuroprotection (PENUT) Trial. *EBioMedicine* **2021**, *72*, 103605.
52. Leviton, A.; Fichorova, R.N.; O'Shea, T.M.; Kuban, K.; Paneth, N.; Dammann, O.; Allred, E.N.; ELGAN Study Investigators. Two-hit model of brain damage in the very preterm newborn: Small for gestational age and postnatal systemic inflammation. *Pediatr. Res.* **2013**, *73*, 362–370.

Supplementary Table S4. Methodological quality of the present systematic review according to the AMSTAR-2 tool

Item	AMSTAR-2 domain	Critical domain	Rating*	Comment specific to this review
1	Research question and inclusion criteria include PICO/PCC elements	No	Yes	The review question and eligibility criteria specify the population (preterm neonates), the concept (inflammatory biomarkers, acute-phase proteins, astroglial injury markers and neurotrophic factors measured in blood, urine or saliva) and the context (prediction of motor/neuropsychomotor development up to 24 months of corrected age).
2	Protocol registered before commencement of the review	Yes	Yes	The methods were established a priori and the protocol was prospectively registered in PROSPERO (CRD42022365839). The Methods section states that no major deviations from the registered protocol occurred.
3	Justification for the selection of study designs	No	Yes	The review focuses on observational cohort studies in which preterm infants are followed over time to assess motor development. The Introduction/Methods explicitly justify the inclusion of cohort designs as the most appropriate approach for this prognostic question, given that inflammatory exposures cannot be randomised for ethical reasons.
4	Comprehensive literature search strategy	Yes	Yes	A comprehensive search was conducted in MEDLINE (via PubMed), Embase, Web of Science, SciELO and the Cochrane Library, without language or date restrictions. Reference lists were also screened. Complete, copy-pasteable search strings and all filters for each database are presented in Supplementary Table S2.
5	Study selection performed in duplicate	No	Yes	After removal of duplicates, three reviewers independently screened titles/abstracts and full texts, with disagreements resolved by a fourth reviewer, as described in the Study selection subsection.
6	Data extraction performed in duplicate	No	Yes	Data extraction was performed independently by three reviewers using a piloted form, and discrepancies were resolved by a fourth reviewer, as stated in the Data extraction subsection.
7	List of excluded studies and justification for exclusions	Yes	Yes	All 22 full-text articles excluded after eligibility assessment are listed in Supplementary Table S3, together with the main reason for exclusion (for example, wrong population, absence of biomarker measurement, no motor outcome).

8	Description of included studies in adequate detail	No	Yes	Study characteristics are described in detail in Tables 1 and 2, including setting, sample size, gestational age and birth weight distributions, key neonatal morbidities, NICU length of stay, biospecimens, laboratory methods, biomarkers assessed and motor development scales, and are further elaborated in the Results section.
9	Use of a satisfactory technique for assessing risk of bias in individual studies	Yes	Yes	Risk of bias of the cohort studies was assessed independently using the Newcastle–Ottawa Scale (NOS), covering selection, comparability and outcome domains. Domain-level ratings, total NOS scores (0–9) and overall quality classifications (high, moderate or low) are presented in Table 1 and described in the Methods and Results sections.
10	Reporting of funding sources for the studies included in the review	No	No	Funding sources and potential industry sponsorship were clearly reported for the present review; however, the funding of individual primary studies was not systematically extracted or summarised across the included cohorts.
11	Appropriate methods for statistical combination of results (if meta-analysis)	Yes	Not applicable	No meta-analysis was performed due to substantial clinical and methodological heterogeneity (different biomarkers, biospecimens, sampling windows and motor/developmental outcomes).
12	Assessment of the potential impact of risk of bias on the results of the synthesis	No	Not applicable	Not applicable, as no quantitative synthesis or meta-analysis was conducted.
13	Consideration of risk of bias when interpreting/discussing the results	Yes	Yes	The Discussion explicitly considers the NOS ratings (high vs moderate quality), limited adjustment for confounding and incomplete follow-up, and states that these limitations reduce confidence in the magnitude and consistency of associations, so the findings should be interpreted as hypothesis-generating rather than confirmatory.
14	Satisfactory explanation of and discussion of any heterogeneity observed	No	Yes	The Discussion examines potential sources of heterogeneity, including differences in gestational age and birth weight, burden of neonatal morbidities, timing and type of biospecimens, laboratory platforms and motor assessment tools, and uses these factors to explain divergent results across cohorts.
15	Investigation and discussion of publication bias (if quantitative synthesis)	Yes	Not applicable	Formal assessment of publication bias was not applicable because fewer than 10 studies were included and no meta-analysis was conducted.

16	Reporting of potential conflicts of interest and funding for the review	No	Yes	The review reports its funding source (CAPES, Finance Code 001) and clearly states that the authors have no competing interests in the Funding and Conflicts of interest sections.

*Rating options according to AMSTAR-2: Yes, Partial yes, No, Not applicable.

Overall AMSTAR-2 rating: high confidence in the results of the review (no critical weaknesses and one non-critical weakness related to the lack of systematic extraction of funding sources from primary studies).

Supplementary Table S5 – Candidate biomarker domains relevant to neonatal brain injury and potential clinical utility (contextual background)

Mechanistic domain	Biomarker (examples)	Common specimen(s)	What it reflects (brief)	Potential clinical/research utility	Availability/notes
Systemic inflammation / immune activation	IL-6, IL-8, TNF-α, IL-1β, IL-10; CRP	Blood (serum/plasma), cord blood; occasionally urine/saliva	Pro-/anti-inflammatory signaling; acute-phase response	Risk enrichment for follow-up; phenotype inflammatory burden; adjunct to clinical risk models	Some (CRP) routine; cytokines often research/ELISA or multiplex; values not comparable across platforms<br>Key references: [24,25,53]
Astroglial / neuronal injury	GFAP; S100B; NSE; UCH-L1; neurofilament light chain (NfL); tau	Blood; CSF (when available); urine in some contexts	Glial/neuronal structural injury and axonal damage	Adjunct to identify/monitor CNS injury severity; research endpoints; may complement neuroimaging	Mostly research/selected centers; neonatal reference ranges and cut-offs still evolving<br>Key references: [54–57]
Neurotrophic / plasticity pathways	BDNF; GDNF; NGF; IGF-1 (context-dependent)	Blood; cord blood; occasionally urine/saliva	Neuroplasticity, neuronal survival, synaptic maturation	Hypothesis-generating prognostic signals; mechanistic insight; potential targets for future panels	Primarily research assays; limited longitudinal evidence in preterm cohorts<br>Key references: [58,59]
Cellular stress response	Heat shock protein 70 (HSP70)	Blood; CSF; saliva (research)	Stress-response and chaperone activity; cellular injury response	Candidate biomarker to explore alongside neuroinjury/inflammation panels	Research; evidence and thresholds for neonatal prognostication are not established<br>Key references: [60–63]
Oxidative / nitrosative stress	F2-isoprostanes; malondialdehyde (MDA); 8-OHdG;	Blood; urine (common for	Lipid/DNA/protein oxidation and antioxidant defenses	Candidate domain for mechanistic phenotyping and prediction models;	Mostly research; assay heterogeneity is

	nitrotyrosine; protein carbonyls; total antioxidant capacity; GSH/GSSG; SOD, catalase, GPx	8-OHdG/isoprostanes); saliva (research)		may relate to white-matter vulnerability windows	substantial<br>Key references: [64,65]
Energy metabolism / mitochondrial dysfunction	Lactate; pyruvate; lactate:pyruvate ratio; acylcarnitines; cytochrome-c (research)	Blood; CSF (selected); urine (metabolomics)	Anaerobic metabolism, mitochondrial impairment, metabolic stress	Monitoring illness severity; potential adjunct domain for multimodal prognostic panels	Lactate is routine; others largely research/metabolomics<br>Key references: [66–68]
Endothelial dysfunction / vascular dysregulation	ICAM-1; VCAM-1; E-selectin; endothelin-1; von Willebrand factor; angiopoietin-2; VEGF; ADMA	Blood (serum/plasma)	Endothelial activation, vascular permeability, microvascular injury	Candidate domain for risk stratification in systemic illness; may complement inflammation panels	Typically research or specialized assays; neonatal norms vary<br>Key references: [69]

Note: This table is provided as contextual background and future research directions; it does not imply clinical validity for motor outcome prediction in preterm infants.

References (Table S5):

24. Dammann, O.; Leviton, A. Intermittent or sustained systemic inflammation and the preterm brain. *Pediatr. Res.* **2014**, *75*, 376–380. https://doi.org/10.1038/pr.2013.238.
25. Reiss, J.D.; Peterson, L.S.; Nesamoney, S.N.; Chang, A.L.; Pasca, A.M.; Marić, I.; Shaw, G.M.; Gaudilliere, B.; Wong, R.J.; Sylvester, K.G.; et al. Perinatal infection, inflammation, preterm birth, and brain injury: A review with proposals for future investigations. *Exp. Neurol.* **2022**, *351*, 113988. https://doi.org/10.1016/j.expneurol.2022.113988.
53. Kuban, K.C.K.; O'Shea, T.M.; Allred, E.N.; Paneth, N.; Hirtz, D.; Fichorova, R.N.; Leviton, A. Systemic inflammation and cerebral palsy risk in extremely preterm infants. *J. Child Neurol.* **2014**, *29*, 1692–1698. https://doi.org/10.1177/0883073813513335.
54. Douglas-Escobar, M.; Heaton, S.C.; Bennett, J.; Young, L.J.; Glushakova, O.; Xu, X.; Barbeau, D.Y.; Rossignol, C.; Miller, C.; Old Crow, A.M.; et al. UCH-L1 and GFAP Serum Levels in Neonates with Hypoxic-Ischemic Encephalopathy: A Single Center Pilot Study. *Front. Neurol.* **2014**, *5*, 273. https://doi.org/10.3389/fneur.2014.00273.
55. Douglas-Escobar, M.; Weiss, M.D. Biomarkers of Brain Injury in the Premature Infant. *Front. Neurol.* **2012**, *3*, 185. https://doi.org/10.3389/fneur.2012.00185.

56. Serpero, L.D.; Pluchinotta, F.; Gazzolo, D. The clinical and diagnostic utility of S100B in preterm newborns. *Clin. Chim. Acta* **2015**, *444*, 193–198. https://doi.org/10.1016/j.cca.2015.02.028.
57. Gaulee, D.; Yang, Z.; Sura, L.; Xu, H.; Rossignol, C.; Weiss, M.D.; Bliznyuk, N. Concentration of Serum Biomarkers of Brain Injury in Neonates With a Low Cord pH With or Without Mild Hypoxic-Ischemic Encephalopathy. *Front. Neurol.* **2022**, *13*, 934755. https://doi.org/10.3389/fneur.2022.934755.
58. Krey, N.; Stocchero, B.A.; Creutzberg, K.C.; Heberle, B.A.; Tractenberg, S.G.; Xiang, L.; Wei, W.; Kluwe-Schiavon, B.; Viola, T.W. Neurotrophic Factor Levels in Preterm Infants: A Systematic Review and Meta-Analysis. *Front. Neurol.* **2021**, *12*, 643576. https://doi.org/10.3389/fneur.2021.643576.
59. Hellström, W.; Hortensius, L.M.; Löfqvist, C.; Hellgren, G.; Tataranno, M.L.; Ley, D.; Benders, M.J.; Hellström, A.; Björkman–Burtscher, I.M.; Heckemann, R.A.; et al. Postnatal serum IGF-1 levels associate with brain volumes at term in extremely preterm infants. *Pediatr. Res.* **2023**, *93*, 666–674. https://doi.org/10.1038/s41390-022-02134-4.
60. Kim, J.Y.; Yenari, M.A. The immune modulating properties of the heat shock proteins after brain injury. Anat. Cell Biol. 2013, 46, 1–7. https://doi.org/10.5115/acb.2013.46.1.1.
61. Kim, J.Y.; Barua, S.; Huang, M.Y.; Park, J.; Yenari, M.A.; Lee, J.E. Heat Shock Protein 70 (HSP70) Induction: Chaperonotherapy for Neuroprotection after Brain Injury. *Cells* **2020**, *9*, 2020. https://doi.org/10.3390/cells9092020.
62. Matsumori, Y.; Hong, S.M.; Aoyama, K.; Fan, Y.; Kayama, T.; Sheldon, R.A.; Vexler, Z.S.; Ferriero, D.M.; Weinstein, P.R.; Liu, J. Hsp70 overexpression sequesters AIF and reduces neonatal hypoxic/ischemic brain injury. J. Cereb. Blood Flow Metab. 2005, 25, 899–910. https://doi.org/10.1038/sj.jcbfm.9600080.
63. Seo, K.; Hwang-Bo, S.; Im, S.A.; Kim, M.; Youn, Y.A. Predictive Value of Heat-Shock Protein Gene Expression on Severe Neonatal Hypoxic-Ischemic Encephalopathy. *Diagnostics* **2022**, *12*, 981. https://doi.org/10.3390/diagnostics12040981.
64. Lembo, C.; Buonocore, G.; Perrone, S. Oxidative Stress in Preterm Newborns. *Antioxidants* **2021**, *10*, 1672. https://doi.org/10.3390/antiox10111672.
65. Perrone, S.; Laschi, E.; Buonocore, G. Biomarkers of oxidative stress in the fetus and in the newborn. *Free. Radic. Biol. Med.* **2019**, *142*, 23–31. https://doi.org/10.1016/j.freeradbiomed.2019.03.034.
66. Ten, V.S.; Stepanova, A.A.; Ratner, V.; Neginskaya, M.; Niatsetskaya, Z.; Sosunov, S.; Starkov, A. Mitochondrial Dysfunction and Permeability Transition in Neonatal Brain and Lung Injuries. *Cells* **2021**, *10*, 569. https://doi.org/10.3390/cells10030569.
67. Shayota, B.J. Biomarkers of mitochondrial disorders. Neurotherapeutics 2024, 21, e00325. https://doi.org/10.1016/j.neurot.2024.e00325
68. Lee, I.C.; Wong, S.H.; Wang, X.A.; Yu, C.S. Identifying Early Diagnostic Biomarkers Associated with Neonatal Hypoxic-Ischemic Encephalopathy. *Diagnostics* **2021**, *11*, 897. https://doi.org/10.3390/diagnostics11050897.
69. Amelio, G.S.; Provitera, L.; Raffaeli, G.; Tripodi, M.; Amodeo, I.; Gulden, S.; Cortesi, V.; Manzoni, F.; Cervellini, G.; Tomaselli, A.; et al. Endothelial dysfunction in preterm infants: The hidden legacy of uteroplacental pathologies. *Front. Pediatr.* **2022**, *10*, 1041919. https://doi.org/10.3389/fped.2022.1041919.
